# De novo characterization of the *Anthurium* transcriptome and analysis of its digital gene expression under cold stress

**DOI:** 10.1186/1471-2164-14-827

**Published:** 2013-11-25

**Authors:** Dan-Qing Tian, Xiao-Yun Pan, Yong-Ming Yu, Wei-Yong Wang, Fei Zhang, Ya-Ying Ge, Xiao-Lan Shen, Fu-Quan Shen, Xiao-Jing Liu

**Affiliations:** Flower research and development center, Zhejiang academy of agricultural sciences, Hangzhou, 311202 China; College of Horticulture, Nanjing Agricultural University, Nanjing, 210095 China; Institute of Botany, Jiangsu Province & Chinese Academy of Sciences, Nanjing, 210014 China

**Keywords:** *Anthurium*, Cold, Transcriptome, Digital gene expression

## Abstract

**Background:**

*Anthurium andraeanum* is one of the most popular tropical flowers. In temperate and cold zones, a much greater risk of cold stress occurs in the supply of *Anthurium* plants. Unlike the freeze-tolerant model plants, *Anthurium* plants are particularly sensitive to low temperatures. Improvement of chilling tolerance in *Anthurium* may significantly increase its production and extend its shelf-life. To date, no previous genomic information has been reported in *Anthurium* plants.

**Results:**

Using Illumina sequencing technology, we generated over two billion base of high-quality sequence in *Anthurium*, and demonstrated *de novo* assembly and annotation of genes without prior genome information. These reads were assembled into 44,382 unigenes (mean length = 560 bp). Based on similarity search with known protein in the non-redundant (nr) protein database, 27396 unigenes (62%) were functionally annotated with a cut-off E-value of 10^-5^. Further, DGE tags were mapped to the assembled transcriptome for gene expression analysis under cold stress. In total, 4363 differentially expressed genes were identified. Among these genes, 292, 805 and 708 genes were up-regulated after 1-h, 5-h and 24-h cold treatment, respectively. Then we mapped these cold-induced genes to the KEGG database. Specific enrichment was observed in photosynthesis pathway, metabolic pathways and oxidative phosphorylation pathway in 1-h cold-treated plants. After a 5-h cold treatment, the metabolic pathways and oxidative phosphorylation pathway were significantly identified as the top two pathways. After 24-h cold treatment, mRNA surveillance pathway, RNA transport pathway and plant-pathogen interaction pathway were significantly enriched. Together, a total of 39 cold-inducible transcription factors were identified, including subsets of AP2/ERF, Zinc figure, NAC, MYB and bZIP family members.

**Conclusion:**

Our study is the first to provide the transcriptome sequence resource for *Anthurium* plants, and demonstrate its digital gene expression profiling under cold conditions using the assembled transcriptome data for reference. These data provides a valuable resource for genetic and genomic studies under abiotic conditions for *Anthurium* plants.

**Electronic supplementary material:**

The online version of this article (doi:10.1186/1471-2164-14-827) contains supplementary material, which is available to authorized users.

## Background

Low temperature is an environmental abiotic stimulus. To adapt to environmental changes, plants have various physiological response and defense systems to withstand chilling and freezing conditions. The regulatory mechanism in higher plants had been analyzed by studying a number of genes responding to cold stress at the transcriptional level [1, 2]. In *Arabidopsis thaliana*, for example, thousands of genes were thought to be involved in abiotic stress [3, 4]. Generally, the cold stress-inducible genes were classified into two groups: one group was that directly protect plant against environmental stresses; and the other was that regulate gene expression network and signaling in stress response [5]. Recent progress had been made in analyzing the functions of stress-inducible genes, not only to understand the mechanisms of cold stress, but also to improve the chilling tolerance of plants by gene transfer. Genetic studies had identified many transcription factors that extensively involved in the regulation network of cold-inducible genes [6]. The most well documented pathways involved a class of DREB/CBF transcription factors, which specific binding to the DRE/CRT cis-elements in the promoters of the target genes [5, 7]. In *A. thaliana*, recent studies also focused on the upstream regulators of DREB/CBF. Several proteins, including ICE1, MYB15 and CAMTA3, had been identified as regulators of DREB1/CBF gene expression [8, 9].

Genome-wide analyses have dramatically improved the efficiency of gene discovery. With the advent of next-generation sequencing, large scale transcriptome data became available in both model species and non-model species [10, 11]. Although microarray technologies continue to make progress, genome-scale studies have been actively pursued for gene discovery and construction of biochemical and regulatory networks [12, 13]. Using the transcriptome data for reference, DGE tag profiling generates a digital output proportional, allowing digital quantification and comparation across different experiments. A number of studies have used high-throughput expression analyses by stress conditions in higher plants. In *A. thaliana*, about 30% of the transcriptome was regulated by abiotic stress, and 2,409 genes were identified of great importance to cold, salt, and drought tolerance [14]. In wheat, the effect of low temperature on transcriptome reprogramming was explored, and over 2% of the wheat genome showed a greater than two-fold changes by cold stress [15]. In roots of cucumber, large-scale differentially expressed genes in several regulatory pathways were identified under waterlogging stress by digital gene expression profile [16]. Although transcriptome changes have been identified, the biochemical functions of many stress-regulated genes remain unknown.

*Anthurium andraeanum* is one of the most popular tropical flowers in markets worldwide. Unlike the freeze-tolerant *A. thaliana*, *Anthurium* plants are irreversibly injured by long-term exposure to temperatures lower than 6°C. In temperate and cold areas, a much greater risk of cold stress occurs in the supply of *Anthurium* plants, especially in the process of production and transportation. After duration of long-time cold exposure, *Anthurium* plants cease to grow, and wilting symptoms begin to appear, with visible signs of yellowing leaves. It has been demonstrated that the physiological changes of cold response is mediated through the differential expression of many genes in model plants [4, 17]. However, little is known about the cold-regulated genes and related pathways in *Anthurium*. To date, no previous genomic information has been reported in *Anthurium* plants, and fewer than 300 ESTs have previously been deposited in Genbank.

In this study, we presented the first comprehensive transcriptome characterization for *Anthurium* plants, and explored the effect of low temperature on global changes in the transcriptome. Using Illumina sequencing technology, we generated over two billion base of high-quality sequence, and demonstrated *de novo* assembly and annotation of genes without prior genome information. Furthermore, we compared the gene expression profiles of cold-treated and control *Anthurium* plants using DGE system, and identified numerous differentially and specifically expressed transcripts of cold-regulated genes. This represents a fully characterized *Anthurium* transcriptome, and provides a valuable resource for genetic and genomic studies of abiotic stress in the future.

## Results

### Sequencing and reads assembly

**Figure 1:**
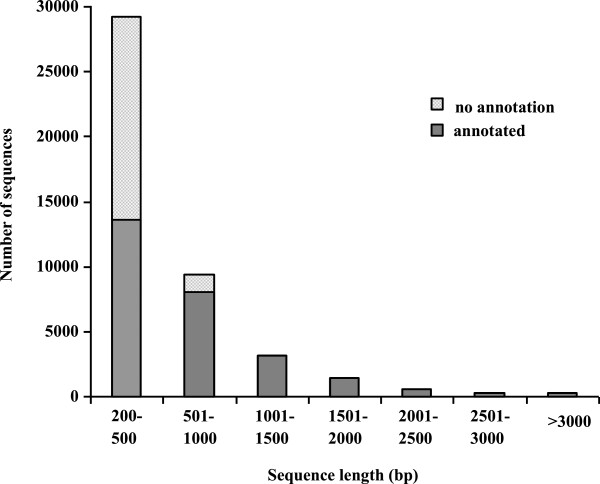
**Histogram of unigene length distributions and the proportion of sequences annotated.** Unigenes were searched in the NCBI nr protien database using blastx with a cut-off E-value of 1.0E^-5^.

To obtain a global overview of *Anthurium* transcriptome, a mixed cDNA sample of seedlings of cold-treated and control plants was prepared and sequenced using Illumina HiSeq™ 2000. Sequencing of normalized cDNA libraries resulted in approximately 2 Gbp of sequence data. After the first assembly, 411,416 contigs were obtained, and the mean contig size was 138 bp with lengths. Then the contigs were further assembled into 73,444 scaffolds with paired-end reads joining. The size distribution of contigs and scaffolds are shown in Additional file 1. After gap fillings of scaffolds, the final assembly consisted of 44,382 unigenes (Table 1). The length distributions of unigenes are given in Figure 1.

**Table 1:** **Sequence statistics of the** ***Anthurium*** **transcriptome**

Total number of reads	23,138,676
Total nucleotides (bp)	2,082,480,840
Total number of contigs	411,416
Mean length of contigs (bp)	138
Total number of scaffolds	73,444
Mean length of scaffolds (bp)	394
Total number of unigenes	44,382
Mean length of unigenes (bp)	560
Sequences with E-value < 10^-5^	27,396

### Functional annotation

**Figure 2:**
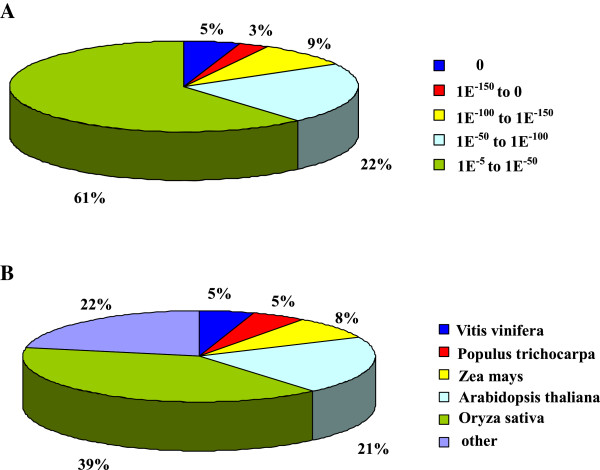
**Characteristics of homology search of query sequences aligned by BLASTx to the nr database. (A)** E-value distribution of unigenes BLASTx hits in the nr database with an E-value threshold of 1.0E^-5^. **(B)** Species distribution of the first BLAST hits for each sequence with a cut-off E-value of 1.0E^-5^.

The unigene sequences were firstly compared to the non-redundant (nr) protein database with a cut-off E-value of 10^-5^. As a result, 27,396 unigenes (62%) were annotated. Figure 1 indicated that the ratio of unigenes that could be matched to known genes had a linear relationship with the length of unigene sequences. As shown in the figure, 98% unigenes over 1,000 bp were annotated with gene names, whereas 46% sequences between 200 to 500 bp could be matched to known proteins (Figure 1). For E-value distribution, 61% homolog sequences ranged between 1.0E^-5^ to 1.0E^-50^, while 39% sequences had a threshold E-value less than 1.0E^-50^ that showed strong homology (Figure 2A). The species that provided the best BLASTx matches (first hit) was *Oryza sativa*, and there were more than ten thousand genes with the highest homology. The next closest species was *A. thaliana*, which showed 21% homology with *Anthurium andraeanum* (Figure 2B).

**Figure 3:**
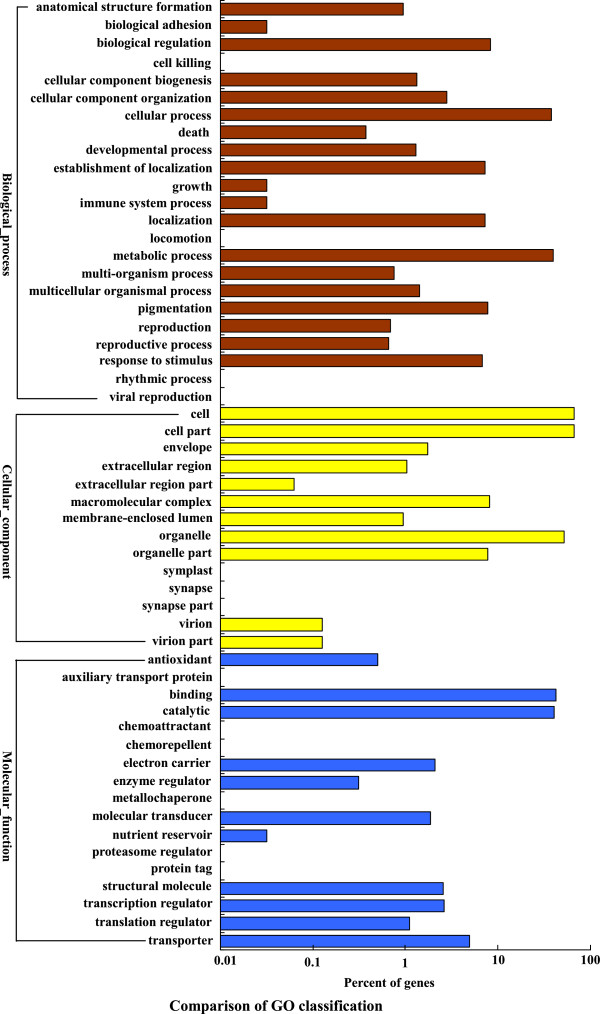
**Histogram of Gene Ontology (GO) classifications.** Biological process (brown), Cellular components (yellow) and Molecular function (blue). Percentages indicate the proportion of unigenes that have GO annotations.

**Figure 4:**
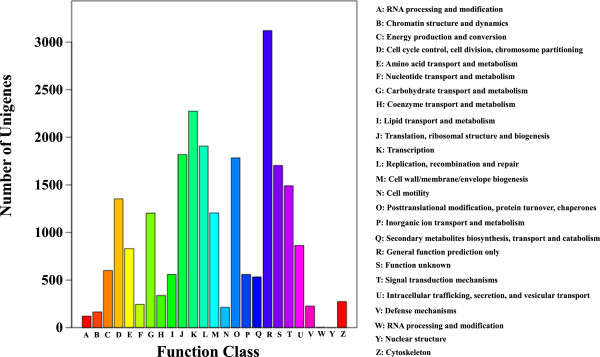
**Distribution of genes in the transcriptome with COG functional classification.** 23367 sequences have a COG classification among 25 categories.

We used Gene Ontology (GO) to classify functions of the annotated genes. Using Blast2GO suite, 13,705 sequences (31%) were categorized to three different GO trees (biological process, cellular component and molecular function). The three main categories were further classified to 54 functional groups. Seven groups, ‘cellular process’, ‘metabolic process’, ‘cell’, ‘cell part’, ‘organelle’, ‘binding’ and ‘catalytic’ are dominant clusters in GO classification (Table 2; Figure 3). We further performed phylogenetic classification using clusters of Orthologous Groups (COG) database. In total, 23,367 genes were matched, and they were grouped into 25 functional classes. The cluster for ‘General function prediction only’ (3,121) and ‘Transcription’ (2,276) were the two largest groups in percentage, which represent 13% and 10% respectively (Table 2; Figure 4). To make further understanding of the transcriptome data, we carried out pathway analysis with Kyoto Encyclopedia of Genes and Genomes (KEGG) mapping of the *Anthurium andraeanum* transcriptome. Totally 14,105 sequences were identified with pathway annotation, and they were functionally assigned to 125 KEGG pathways. The ‘metabolic pathways’ contributed to the greatest parts (3,473 members, 24.62%), followed by ‘spliceosome’ (1,320 members, 9.36%) and ‘plant-pathogen interaction’ (1,180 members, 8.37%) (Table 2; Additional file 2).

**Table 2:** **All-in-one list of** ***Anthurium*** **transcriptome annotations**

Total unigenes	44,382	100%
Nr (E-value < 10^−5^)	27,396	61.73%
Swissprot	19,522	43.99%
COG	23,367	52.65%
KEGG	14,105	31.78%
GO	13,705	30.88%

### Digital gene expression (DGE) library sequencing

**Figure 5:**
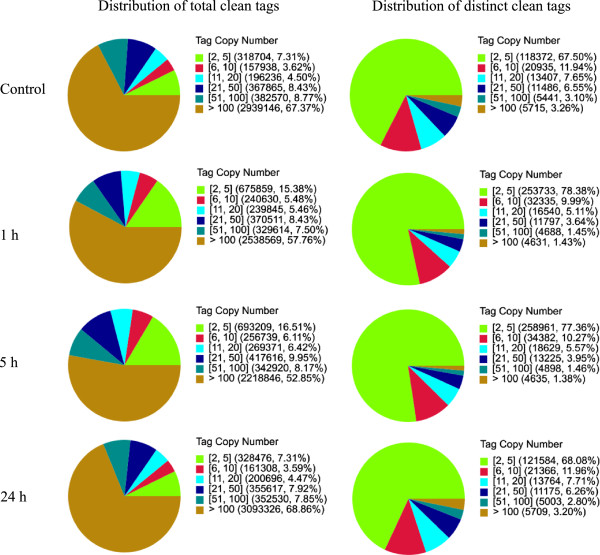
**Distribution of total tags and distinct tags of DGE sequencing.** The left figure is the distribution of total tag number, and the right figure is the distribution of distinct clean tags. Numbers in the brackets indicates the range of copy numbers.

To generate digital expression signatures for *Anthurium* plants under low temperature, we used Solexa (Illumina) technology for sequencing. Briefly, using DGE technology, we sequenced six libraries: control plant samples before transferred from 23 to 6°C (warm sample), and cold-treated samples at 1, 2, 5, 10, 24 h under 6°C after transfer. To facilitate our analysis, three cold-treated samples (1, 5 and 24 h) were selected for comparation with control plants in the following analysis. In total, the DGE libraries generated between 4.6 and 4.9 million raw reads. After removing low quality reads, total number of clean tags per library ranged from 4.2 to 4.5 million, and number of clean tags entitles with unique nucleotide sequences ranged from 1,327,437 to 2,008,977 (Table 3). To evaluate the normality of the DGE data, we performed analysis of the distribution of tag expression. As shown in Figure 5, the distribution of total tags and distinct clean tags over different tag-abundance categories displayed similar patterns for all four DGE libraries. Small number categories of mRNA had high abundance, while the rest majority stays at a very low level of expression.

**Table 3:** **Statistics of DGE sequencing of** ***Anthurium*** **leaves under low temperature**

Summary		Control	1 h	5 h	24 h
Raw data	Total	4,755,703	4,867,906	4,600,240	4,886,350
Raw data	Distinct tag	559,286	787,594	729,441	563,310
Clean tag	Total number	4,362,459	4,395,028	4,198,701	4,491,953
Clean tag	Distinct tag number	175,356	323,724	334,730	178,601
All tag mapping to gene	Total number	1,914,702	1,580,825	1,327,437	2,008,977
All tag mapping to gene	Total % of clean tag	43.89%	35.97%	31.62%	44.72%
All tag mapping to gene	Distinct tag number	51,341	41,139	57,996	49,116
All tag mapping to genee	Distinct tag % of clean tag	29.28%	12.71%	17.33%	27.50%
Unambiguous tag mapping to gene	Total number	1,906,521	1,569,945	1,315,834	2,001,497
Unambiguous tag mapping to gene	Total % of clean tag	43.70%	35.72%	31.34%	44.56%
Unambiguous tag mapping to gene	Distinct Tag number	51,026	40,805	57,541	48,784
Unambiguous tag mapping to gene	Distinct Tag % of clean tag	29.10%	12.60%	17.19%	27.31%
All tag-mapped genes	number	19,417	17,817	21,352	19,376
All tag-mapped genes	% of ref genes	43.75%	40.14%	48.11%	43.66%
Unambiguous tag-mapped Genes	number	19,287	17,674	21,195	19,217
Unambiguous tag-mapped Genes	% of ref genes	43.46%	39.82%	47.76%	43.30%
Unknown tag	Total number	2,447,757	2,814,203	2,871,264	2,482,976
Unknown tag	Total % of clean tag	56.11%	64.03%	68.38%	55.28%
Unknown tag	Distinct Tag number	124,015	282,585	276,734	129,485
Unknown tag	Distinct Tag % of clean tag	70.72%	87.29%	82.67%	72.50%

### Mapping DGE sequences to the reference transcriptome database

To reveal the molecular events of the DGE profiles, application of the transcriptome data was convened into further study of its digital gene expression signatures under low temperature in *Anthurium* plants. We mapped the tag sequences of the four DGE libraries to our transcriptome reference database. The reference database contained 44,382 unigenes. Among the generated distinct tags, 41,139 to 57,996 distinct tags were mapped to a gene in the reference transcriptome database. Over 40% sequences in the reference tag database could be mapped to a unique tag (Table 3). The saturation analysis was performed to check whether the number of detected genes increased proportionally to the sequence amount. Additional file 3 showed the trend of saturation, and the number of detected genes almost ceased to increase when sequencing amount reached 2 million or higher. The gene expression level was determined by number concentrations using TPM (number of transcripts per million clean tags). The normalized final counts were measured. As summarized in Additional file 4, only a small number of genes were highly expressed, most of the genes had fewer than 10 copies.

### Analysis of differential gene expression

**Figure 6:**
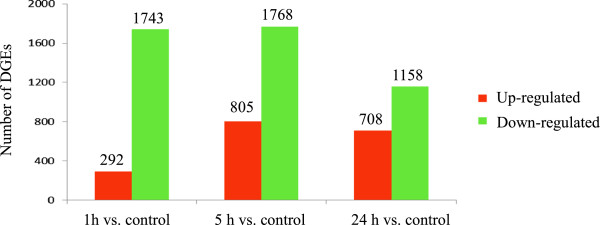
**Differentially expressed genes (DEGs) for** ***Anthurium*** **leaves under low temperature.** The number of up-regulated and down-regulated genes in cold treated plants was shown. We use “FDR (False Discovery Rate) ≤ 0.001 and the absolute value of log2Ratio ≥ 1” as the threshold to judge the significance of gene expression difference. Control plants were grown at 23°C. 1 h, 5 h, 24 h, plants were treated under 6°C low temperature for 1 h, 5 h and 24 h, respectively.

A total of 4,363 significantly changed genes were detected between cold-treated and control libraries. We found that over 50% of the differentially expressed genes had no homologues in the NCBI database. Screening of differentially expressed genes, the results showed that both up- and down-regulation of gene expression occur, but that, the expression of genes changed over time of cold treatments. Among all differentially expressed genes, 292 were quickly induced by cold while 1,743 were down-regulated at 1 h (Figure 6; Additional file 5). After 5-h cold treatment, 805 genes were up-regulated, and 1,768 were down-regulated (Figure 6; Additional file 6). After 24-h cold stress treatment, 708 genes were up-regulated, and 1,158 were down-regulated (Figure 6; Additional file 7).

### Metabolic pathway by KEGG analysis of cold up-regulated genes

In this study, we concentrated on up-regulated genes by cold stress, and genes with expression ratios greater than 2 times compared with unstressed plants were defined as cold-inducible genes. To understand the biological function of these genes, we mapped the cold-induced genes to terms in KEGG database, with a view to identifies significantly enriched metabolic pathways or signal transduction pathways in DEGs comparing with the whole genome background. Among the mapped pathways, six pathway were significantly enriched (Qvalue ≤ 0.05) after the 1-h cold treatment. Notably, specific enrichment was observed in photosynthesis pathway, metabolic pathways and oxidative phosphorylation pathway in 1-h cold-treated plants. After a 5-h cold treatment, the metabolic pathways and oxidative phosphorylation pathway were significantly identified as the top two pathways. After 24-h cold treatment, we found that transcripts involved in mRNA surveillance pathway, RNA transport pathway and plant-pathogen interaction pathway were significantly enriched (Table 4).

**Table 4:** **Significantly enriched pathways of up-regulated genes by cold stress in** ***Anthurium***

	Pathway	Number of DEGs with pathway annotation (226)	Number of all genes with pathway annotation (14105)	Qvalue	Pathway ID
1 h vs. control	Photosynthesis	13 (15.85%)	61 (0.43%)	6.40e-16	ko00195
	Metabolic pathways	45 (54.88%)	3594 (25.48%)	2.41e-07	ko01100
	Oxidative phosphorylation	11 (13.41%)	216 (1.53%)	5.76e-07	ko00190
	RNA polymerase	13 (15.85%)	558 (3.96%)	1.75e-04	ko03020
	Pyrimidine metabolism	14 (17.07%)	672 (4.76%)	2.24e-04	ko00240
	Purine metabolism	14 (17.07%)	729 (5.17%)	4.36e-04	ko00230
5 h vs. control	Metabolic pathways	122 (36.31%)	3594 (25.48%)	4.77e-04	ko01100
	Oxidative phosphorylation	16 (4.76%)	216 (1.53%)	2.58e-04	ko00190
	mRNA surveillance pathway	32 (9.52%)	669 (4.74%)	3.58e-03	ko03015
	Arginine and proline metabolism	10 (2.98%)	103 (0.73%)	3.58e-03	ko00330
	RNA polymerase	27 (8.04%)	558 (3.96%)	5.58e-03	ko03020
	RNA transport	36 (10.71%)	834 (5.91%)	5.58e-03	ko03013
	Ascorbate and aldarate metabolism	8 (2.38%)	77 (0.55%)	5.77e-03	ko00053
	Photosynthesis	7 (2.08%)	61 (0.43%)	6.21e-03	ko00195
	Pyrimidine metabolism	29 (8.63%)	672 (4.76%)	1.38e-02	ko00240
	Pyruvate metabolism	11 (3.27%)	182 (1.29%)	3.60e-02	ko00620
	Purine metabolism	29 (8.63%)	729 (5.17%)	3.65e-02	ko00230
24 h vs. control	mRNA surveillance pathway	31 (13.72%)	669 (4.74%)	9.61e-06	ko03015
	RNA transport	31 (13.72%)	834 (5.91%)	1.02e-03	ko03013
	Plant-pathogen interaction	39 (17.26%)	1180 (8.37%)	1.06e-03	ko04626

(Pathways with Qvalue ≤ 0.05 are significantly enriched in DEGs).

### Analysis of cold up-regulated genes identified as putative transcription factors

**Figure 7:**
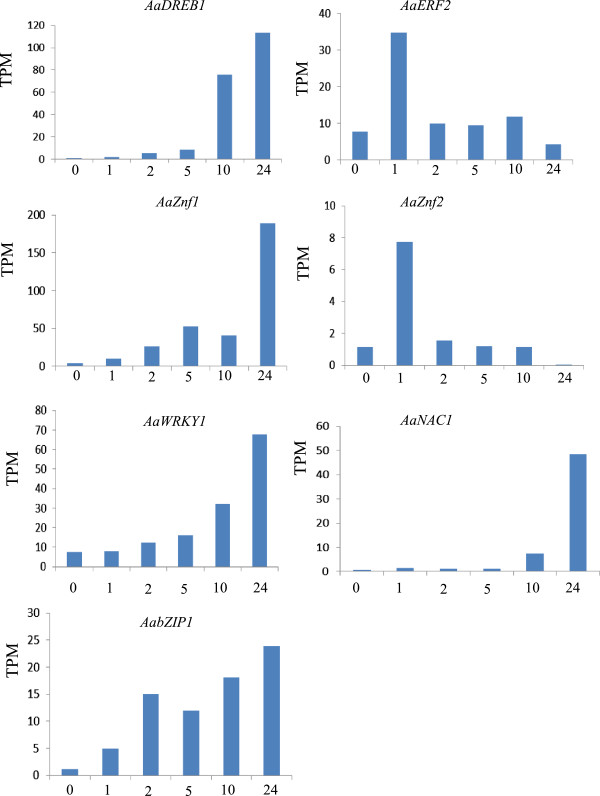
**Analysis of differentially expressed transcription factor genes in** ***Anthurium*** **under low temperature.** The gene expression levels were determined by calculating the number of unambiguous tags for each gene and then normalized to TPM (transcript copies per million tags).

Given that transcription factors appear to have a major effect on the network of cold-responsive genes, an objective of our work was to identify cold-inducible transcription factors. Together, a total of 39 up-regulated genes encode known or putative transcription factors. As shown in Table 5, twelve genes were characterized as AP2/ERF family transcription factors, constituting a large proportion of cold-inducible transcription factors. Among these genes, three dehydration-responsive element binding proteins (DREB) were found to be significantly induced by cold stress in *Anthurium*. A subset of Zinc figure family members, including WRKY subfamily, were involved in responses to cold stimuli. Next, relative small group of NAC, MYB and bZIP transcription factors family members was characterized as cold-inducible transcription factors (Table 5). To further evaluate the role of these transcription factors, we analyzed the expression level of certain transcription factors by calculating the number of unambiguous clean tags and then normalizing to TPM (transcript copies per million clean tags). As shown in Figure 7, these transcription factor genes were significantly up-regulated by cold stress. However, the induction model varied among transcription factor genes. For example, the expression of AP2/ERF1 and Znf1 was induced shortly after starting cold treatment, and the expression increased gradually over the treatment time. AP2/ERF2 and Znf2 had an expression peak at 1 h, and then the expressions decreased quickly to a relative low level.

**Table 5:** **Transcription factors up-regulated by cold stress in** ***Anthurium***

Gene ID	Annotation	TF family	log2 ratio		
			1 h vs. control	5 h vs. control	24 h vs. control
Unigene8754	Dehydration-responsive element binding	AP2/ERF	7.31	**9.74**	**13.47**
Unigene4187	Dehydration-responsive element binding	AP2/ERF	6.09	5.58	**8.48**
Unigene9666	Dehydration-responsive element binding	AP2/ERF	-	**1.33**	0.36
Unigene14111	Ethylene signal	AP2/ERF	-	**8.38**	6.07
Unigene37074	Ethylene-responsive	AP2/ERF	1.2	0.79	**4.07**
Unigene31698	Ethylene-responsive	AP2/ERF	-	-	**2.8**
Unigene33136	AP2 domain	AP2/ERF	-	**2.37**	-
Unigene9720	ERF domain-containing	AP2/ERF	**2.16**	0.29	-
Unigene14276	AP2/ERF	AP2/ERF	0.48	-	**1.85**
Unigene37112	AP2/ERF	AP2/ERF	-	-	**1.25**
Unigene2974	AP2-like	AP2/ERF	-	-	0.75
Unigene40903	Ethylene-responsive	AP2/ERF	-	-	**1.25**
Unigene32148	Zinc finger	Znf	**3.39**	**2.2**	**3.99**
Unigene15996	Zinc finger	Znf	1.42	**3.76**	**5.6**
Unigene8801	NF-X1-type Zinc finger	Znf	0.18	-	**2.23**
Unigene14390	Zinc finger	Znf	6.51	5.58	**10.23**
Unigene37357	RING-H2 finger	Znf (RING-finger)	**2.79**	0.86	1.12
Unigene2924	RING-H2 finger	Znf (RING-finger)	**2.75**	0.05	-
Unigene29742	RING-H2 finger	Znf (RING-finger)	1.06	0.57	0.61
Unigene42394	Zinc knuckle family	Zinc knuckle	-	-	0.76
Unigene37690	WRKY	Znf (WRKY)	6.09	7.58	**11.09**
Unigene12401	WRKY	Znf (WRKY)	0.07	1.1	**3.17**
Unigene32684	WRKY	Znf (WRKY)	-	5.58	**8.56**
Unigene38336	WRKY	Znf (WRKY)	0.99	0.46	**3.32**
Unigene18028	WRKY	Znf (WRKY)	-	-	**1.99**
Unigene22490	NAC domain	NAC	-	8.03	**8.65**
Unigene40941	NAC protein	NAC	0.99	0.79	**6.14**
Unigene27064	NAC protein	NAC	-	1.14	**3.43**
Unigene10069	NAM containing	NAC	-	-	**1.33**
Unigene41065	MYB	MYB	0.4	**2.92**	1.37
Unigene26784	MYB	MYB	-	-	**8.29**
Unigene38186	R2R3 MYB	MYB	-	**2.69**	-
Unigene37949	bZIP	bZIP	2.12	**3.37**	**4.37**
Unigene3240	bZIP domain class	bZIP	0.89	**1.14**	0.91
Unigene5342	Heat stress	HSF	-	**1.6**	**2.03**
Unigene40194	Heat stress	HSF	-	6.57	**8.38**
Unigene40054	GRAS family	GRAS	0.17	0.05	**1.24**
Unigene37810	WHY1 (WHIRLY 1)	PBF-2/Whirly	-	**2.11**	-
Unigene33457	Auxin responsive	Aux/IAA	-	**8.38**	-

Significant difference (FDR ≤ 0.001 and the absolute value of log2Ratio ≥ 1) in relative levels were shown in boldface. FDR, False Discovery Rate. log2 Ratio, log fold changes using the ratio base 2 logarithm.

## Discussion

### Evaluation of *de novo* transcriptome assembly quality

Despite the great advances in DNA sequencing technologies, no genomic data is available to date for *Anthurium* plants. With the development of RNA-seq, transcriptome analysis has become an attractive alternative for in-depth analysis at high resolution. In this study, we carried out *de novo* transcriptome assembly using short-read (Illumina) sequencing. To date, there are many studies using the Roche 454 GS platform [18, 19], which has relative longer reads but at the expense of less sequence data per run. However, the Illumina generates greater depth of sequencing, ensuring more complete coverage of the transcriptome comparing with 454 GS platform. In this study, despite using shorter reads, our assembly is comparable to other published transcriptomes using 454 GS platform. As shown in Figure 1, more than13% unigenes were greater than 1 kb, and 34% unigenes were greater than 500 bp. These results demonstrated the effectiveness of the assembly in capturing a large portion of the transcriptome. Another useful metric is the proportion of the unigenes and its corresponding BLAST hit. Due to lack of genomic resources for *Anthurium*, the proportions of unigenes that have significant similarity to known proteins in GenBank were considered as ‘gold standard’ reference in our studies. Nearly 61.73% of our unigenes had matches in nr protein database, and this value was as high as other comparable statistics reported in *de novo* assemblies.

### Analysis of differentially expressed genes

It has long been known that extensive changes of gene expression occur when plants are exposed to cold stress [2]. Generally, both up- and down-regulation of gene expression occur under cold conditions. It was reported that more genes were up-regulated than down-regulated under cold stress in model plants [15]. In *A. thaliana*, it was reported that 302 genes were found to be cold responsive, and 88 (27%) decreased in abundance [20]. In wheat, over 2% of the whole genome showed altered levels of expression in response to cold stress. Among these genes, 1,711 genes were induced by a cold shock, and 1,402 were down-regulated [15]. In this study, 4,363 differently expressed genes were identified in *Anthurium*, and the relative genomic proportion devoted to cold stress is unknown due to the lack of genome resources. About 30% genes were found to be cold inducible, more than 70% decreased in abundance. These results suggested that plants responses vary in their abilities to adapt to cold stress. Gene expression in tropical plants might extensively differ from that in freeze-tolerant model plants conferring their response to cold stimuli. Among the differentially expressed genes regulated by cold stress in *Anthurium*, over 50% of them had no homologues in the NCBI database. Some of these genes might represent new cold-responsible transcripts which have not been reported in model plants.

### Photosystems

Photosynthesis is highly sensitive to changing temperatures. In barley, it has been reported that light and photosynthetic activity play an important role in plant frost resistance under cold conditions [21]. In meadow fescue, about 50% of the differentially changed proteins were involved in photosynthesis during cold conditions [4]. In this study, to understand the biological function of the genes in *Anthurium*, we mapped the differentially expressed genes to terms in KEGG database. The result showed that photosynthesis pathway was significantly enriched after 1-h cold treatment, suggesting a role in early response to low temperature. Detailed information of photosynthesis pathway in KEGG database indicated that photosystems I and II (PSI and PSII) were rapidly influenced by cold stress (see Additional file 8). The electrons from PSI and PSII might transfer to oxygen, and thereby leads to substantial generation of reactive oxygen species. Enrichment of oxidative phosphorylation pathway after 1-h cold treatment suggested that light-induced ROS production might occur. However, this hypothesis still lack of experimental evidence.

### Major transcription factors of cold stress response

Plants have evolved physiological and biochemical ways in order to cope with cold stress. These responses require expression of large-scale cold-responsive genes. In model plants, transcription factors have been described to control the network of many target genes through direct binding to cis-elements of gene promoter regions [22–24]. In *Anthurium*, 39 up-regulated genes were identified as putative transcription factors, including AP2/ERF, Zn-finger, WRKY, NAC, MYB, bZIP families. These transcription factors exhibited different induction patterns over time periods (Table 5; Figure 7). Based on our results of *Anthurium* plants, the ‘early rapid response’ (phase-1) to cold stress might occurred within the initial 1 hour, while the ‘early slow response’ (phase-2) occurred between 1 and 5 hours. Majority of cold up-regulated transcription factors were activated at phase-1 and phase-2, and the transcription factors activated at phase-1 might play a key role in the activation of the coordinated expression of down-stream genes. Fewer transcription factors exhibited ‘late response’ profiles (phase-3) until 24 hours, and these transcription factors were likely controlled by phase-1 transcription factors.

One group of the most studied transcription factors involved in cold responses is the AP2/ERF family transcription factors, which includes four major subfamilies: the AP2, ERF, RAV and dehydration-responsive element-binding protein (DREB/CBF) subfamilies [25]. Of these, the DREB subfamily has been described as major factors involved in plant cold-stress responses [26]. The role of DREBs/CBFs has been well established in numerous plants, such as *A. thaliana*[27], rice [28], maize [29] and wheat [15]. The *DREB1s/CBFs* expression levels were positively correlated with cold tolerance and it activate the expression of downstream cold-responsive genes via specific binding to the DRE/CRT cis-acting element in their promoters [6]. In *Anthurium*, three DREB genes were found to be quickly and significantly induced by cold stress. The well-established DREB1/CBF pathway might functionally act in *Anthurium* plants.

Although the DREB1/CBF regulon appeared to be one of the main regulatory pathways involved in cold stress, and multiple low-temperature regulatory pathways in addition to the DREB1/CBF pathway were also studied. In *A. thaliana*, transcriptome-profiling indicated at least 28% of the cold-responsive genes were independent of the CBF cold response pathway, including at least 15 transcription factors [20]. In wheat, at least one-third of the cold-inducible genes were not responsive to CBF regulation [17]. Discovery of novel regulators and regulons by cold stress has always been a continuing story. Recently, several zinc-figure transcription factors were discovered to be novel regulators in *A. thaliana* and rice [30, 31]. A novel MYBS3 transcription factor was identified in rice, and it could repress the well-known DREB1/CBF dependent signaling pathway [32]. In *Anthurium* plants, many transcription factors in addition to AP2/ERF family were actively induced by cold stress. Notably, zinc-figure proteins constitute the largest cold-inducible transcription factor family within the initial 1 hour in *Anthurium* (Table 5). Early activation of these transcription factors implied that certain zinc-figure proteins might acts in parallel to the DREB /CBF pathway. Certain regulatory mechanisms might exist involving these transcription factors and their regulons in cold stress. Further investigation should focus on the role of these transcription factors and the related pathways under abiotic stress.

## Conclusion

In this study, we used high-throughput sequencing data to characterize the transcriptome of *Anthurium*, a species for which little genomic data are available. Further, DGE tags were mapped to the assembled transcriptome for further gene expression analysis. A large number of candidate genes involved in cold stress were identified. This represents a fully characterized transcriptome, and provides a valuable resource for genetic and genomic studies in *Anthurium* plants. Additionally, DGE profiling provides new leads for functionally studies of genes involved in abiotic stress.

## Methods

### Sampling

*Anthurium* cv. Alabama were used as experimental materials. Micropropagated plants were transferred and grown in the greenhouse for three month in Zhejiang academy of agricultural sciences. Control plants were then grown in a growth chamber under continuous light (20–40 μm sec^-1^ m^-2^) at 23°C. For cold treatments, plants were transferred to 6°C under the same light source. Fresh tissues were collected at various periods (1, 2, 5, 10 and 24 h). Samples of stems and leaves were separated and frozen in liquid nitrogen for further analysis.

### RNA extraction and library preparation for tanscriptome

Total RNA was isolated using TRIzol reagent (Invitrogen, Carlsbad, CA) according to the manufacturer's instructions. Poly (A) + mRNA was purified with oligo (dT) beads, and then the mRNA was randomly segmented into small fragments by divalent cations (Fragmentation Buffer, Illumina, Hayward, CA) at 94°C for 5 min. These short fragments were used as templates to synthesize the first-strand cDNA using random hexamer primers. The second-strand cDNA was synthesized using RNaseH and DNA polymerase I. Short cDNA fragments were purified with QiaQuick PCR extraction kit. After that, the cDNA fragments were connected with sequencing adapters according to Illumina’s protocol (San Diego, CA USA). After agarose gel electrophoresis, the target fragments of 300–500 bp were selected for PCR amplification to create the final cDNA library.

### Sequencing, *de novo* assembly and functional annotation

A mixed cDNA sample of control plants and 1, 2, 5, 10 and 24 h cold-treated plants were prepared for sequencing. The library was sequenced using Illumina HiSeq™ 2000. Raw reads were cleaned by removing adaptor sequences. Empty reads and reads with unknown sequences ‘N’ were removed before data analysis. Then *de novo* assembly was carried out with short reads assembling program-SOAPdenovo [33]. Firstly, short reads were combined with certain length of overlap to form longer fragments without N, which were called contigs. Then the reads were mapped back to contigs, and congtigs from the same transcript as well as the distances between these contigs were detected with paired-end reads. Next, SOAPdenovo connected the contigs using N to represent unknown sequences between each two contigs, and then scaffolds were made. Paired-end reads were used again for gap filling of scaffolds to get sequences with least Ns and cannot be extended on either end. The final sequences were defined as unigenes. After assembly, unigenes were compared to the non-redundant (nr) protein database with a cut-off E-value of 10^-5^. The Gene Ontology [34] was analyzed using Blast2go software [35]. In a final step, the COG [36] and KEGG pathway [37] annotation was performed, and the best aligning results were used to decide sequence direction of unigenes. The raw data are available in the ArrayExpress database (http://www.ebi.ac.uk/arrayexpress) under accession number E-MTAB-1955.

### Digital gene expression library preparation and sequencing

Total RNAs were extracted from cold-treated and control plants. About 6 μg of total RNA was prepared, and treated with Oligo (dT) magnetic beads adsorption to purify mRNA. Then the first- and second- strand cDNA were synthesized using Oligo (dT) as a primer. The 5′-ends of tags were generated by restriction enzyme NlaIII, which recognize and cut off the CATG sites. Subsequently, the cDNA fragments connected to Oligo (dT) beads were purified, and the Illumina adaptor 1 was ligated to the 5′ end of the digested cDNA fragments. The junction of Illumina adaptor 1 and CATG site was the recognition site of endonuclease MmeI. MmeI cut at 17 bp downstream of the CATG site, producing tags with adaptor 1. After removal of 3′ fragments with magnetic beads precipitation, Illumina adaptor 2 was introduced to 3′ ends of tags, acquiring 21 bp tags with different adaptors at both ends to form a tag library. After 15 cycles of linear PCR amplification, 105 bp fragments were purified by TBE-polyacrylamide gel electrophoresis. After denaturation, the single-chain molecules were fixed onto the Illumina Sequencing Chip. Each molecule grew into a single-molecule cluster sequencing template through *in situ* amplification. Then four types of nucleotides labeled by four colors were added, and sequencing was performed with the method of sequencing by synthesis (SBS). Each tunnel generated millions of raw reads with sequencing length of 49 bp. The DGE data are available in the ArrayExpress database (http://www.ebi.ac.uk/arrayexpress) under accession number E-MTAB-1955.

### Analysis and mapping of DGE tags

Sequencing-received raw image data was transformed by base calling into sequence data, which was called raw data or raw reads. Raw sequences have 3′ adaptor fragments as well as a few low quality sequences. Therefore, several types of impurities were filtered. The raw sequences were transformed into clean tags by removing adaptor sequences, empty reads and low quality sequences (reads with unknown sequences ‘N’). A virtual database containing all the possible CATG + 17 bases length sequences of the reference gene sequences was created. All clean tags were mapped to the reference sequences and only 1 bp mismatch was considered. Clean tags mapped to reference sequences from multiple genes were filtered. Remainder clean tags were designed as unambiguous clean tags. The number of unambiguous clean tags for each gene was calculated and then normalized to TPM (number of transcripts per million clean tags). A rigorous algorithm was used to identify differentially expressed genes between two samples [38]. FDR (false discovery rate) was used to determine the threshold of P value in multiple analyses. We used “FDR ≤ 0.001 and the absolute value of log2Ratio (cold/control) ≥1” as the threshold to judge the significance of gene expression difference. More stringent criteria with smaller FDR and bigger fold-change value can be used to identify DEGs. For pathway enrichment analysis, we looked for significantly enriched metabolic pathways or signal transduction pathways in DEGs comparing with the whole genome background.

## Electronic supplementary material

Additional file 1: **The size distribution of contigs and scaffolds of Anthurium transcriptome.** (A) Size distribution of Illumina sequencing contigs. (B) Size distribution of scaffolds. (PDF 94 KB)

Additional file 2: **KEGG mapping of the** ***Anthurium*** **transcriptome.** (XLSX 18 KB)

Additional file 3: **Saturation evaluation of different gene expression.** (PDF 133 KB)

Additional file 4: **The gene expression level in DGE libraries.** TPM (Transcripts Per Million clean tags) is a standardized indicator, pointing out number of transcript copies in every 1 million clean tags. The number of unambiguous clean tags for each gene was calculated and then normalized to TPM. (XLSX 261 KB)

Additional file 5: **The differentially expressed genes between 1-h cold treatment and control plants.** TPM: transcript copies per million tags. FDR: false discovery rate. We used “FDR ≤ 0.001 and the absolute value of log2Ratio ≥ 1” as the threshold to judge the significance of gene expression difference. (XLSX 355 KB)

Additional file 6: **The differentially expressed genes between 5-h cold treatment and control plants.** TPM: transcript copies per million tags. FDR: false discovery rate. We used “FDR ≤ 0.001 and the absolute value of log2Ratio ≥ 1” as the threshold to judge the significance of gene expression difference. (XLSX 252 KB)

Additional file 7: **The differentially expressed genes between 24-h cold treatment and control plants.** TPM: transcript copies per million tags. FDR: false discovery rate. We used “FDR ≤ 0.001 and the absolute value of log2Ratio ≥ 1” as the threshold to judge the significance of gene expression difference. (PDF 122 KB)

Additional file 8: **Significantly enriched pathways by 1-h cold stress in** ***Anthurium.*** (A) Significantly enriched pathways of differentially expressed genes by 1-h cold stress in *Anthurium*. (B) Detailed information of photosynthesis pathway in KEGG database. (PDF 248 KB)
