# Supplementary figures and images for: De novo characterization of the Anthurium transcriptome and analysis of its digital gene expression under cold stress
Source: BMC Genomics. 2013 Nov 25;14(1):827. doi: 10.1186/1471-2164-14-827 (PMC4046746; doi:10.1186/1471-2164-14-827)

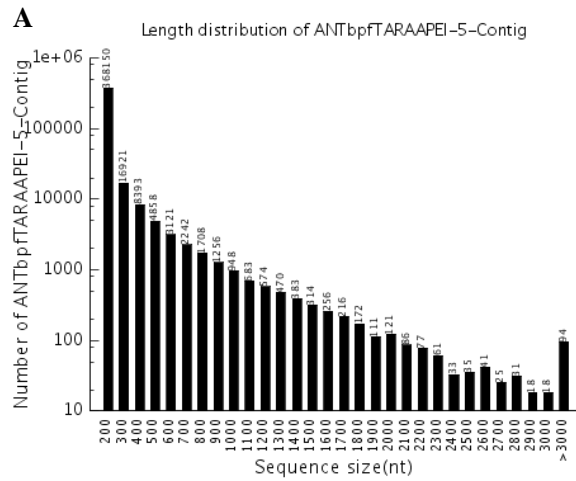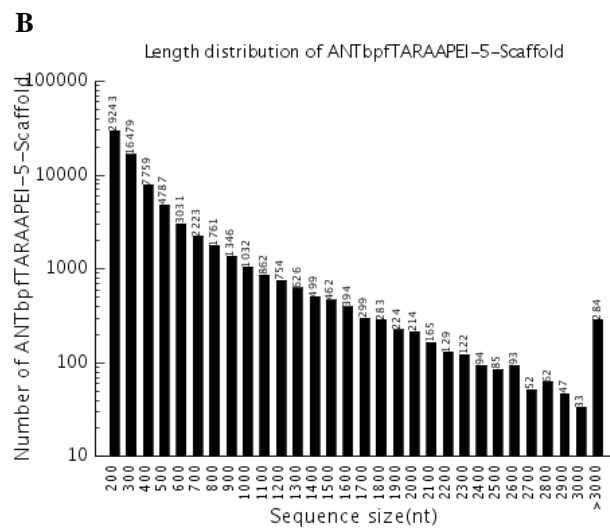

Supplement: Supplementary file 1 — Additional file 1: The size distribution of contigs and scaffolds of Anthurium transcriptome. (A) Size distribution of Illumina sequencing contigs. (B) Size distribution of scaffolds. (PDF 94 KB) [file 12864_2013_5516_MOESM1_ESM.pdf]

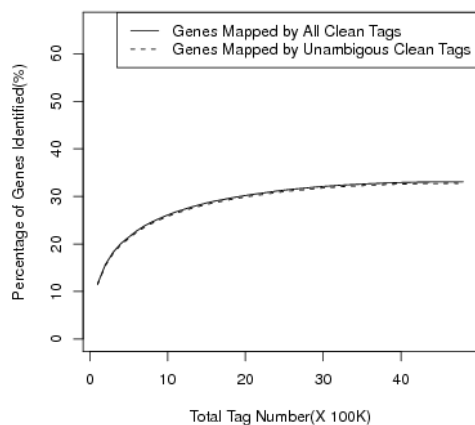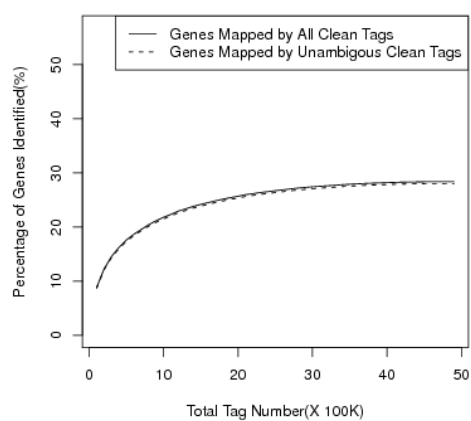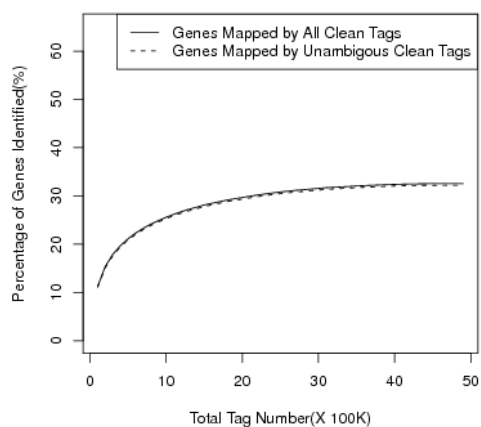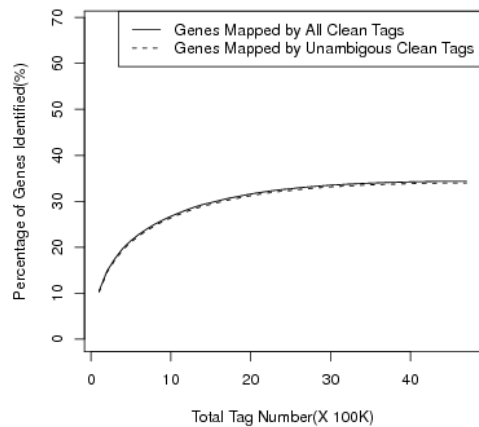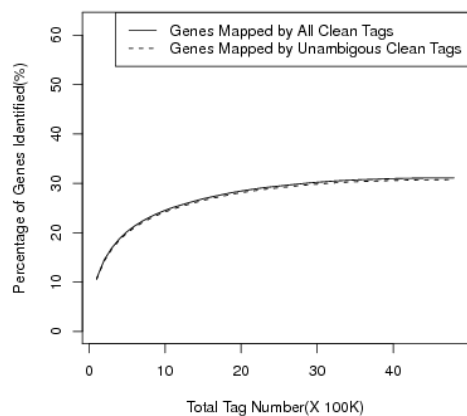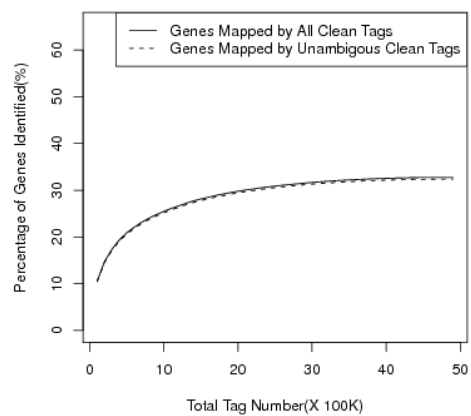

Supplement: Supplementary file 7 — Additional file 7: The differentially expressed genes between 24-h cold treatment and control plants. TPM: transcript copies per million tags. FDR: false discovery rate. We used “FDR ≤ 0.001 and the absolute value of log2Ratio ≥ 1” as the threshold to judge the significance of gene expression difference. (PDF 122 KB) [file 12864_2013_5516_MOESM7_ESM.pdf]
