# Supplementary material for: De novo characterization of the Anthurium transcriptome and analysis of its digital gene expression under cold stress
Source: BMC Genomics. 2013 Nov 25;14(1):827. doi: 10.1186/1471-2164-14-827 (PMC4046746; doi:10.1186/1471-2164-14-827)
Supplement: Supplementary file 3 — Additional file 3: Saturation evaluation of different gene expression. (PDF 133 KB) [file 12864_2013_5516_MOESM3_ESM.pdf]

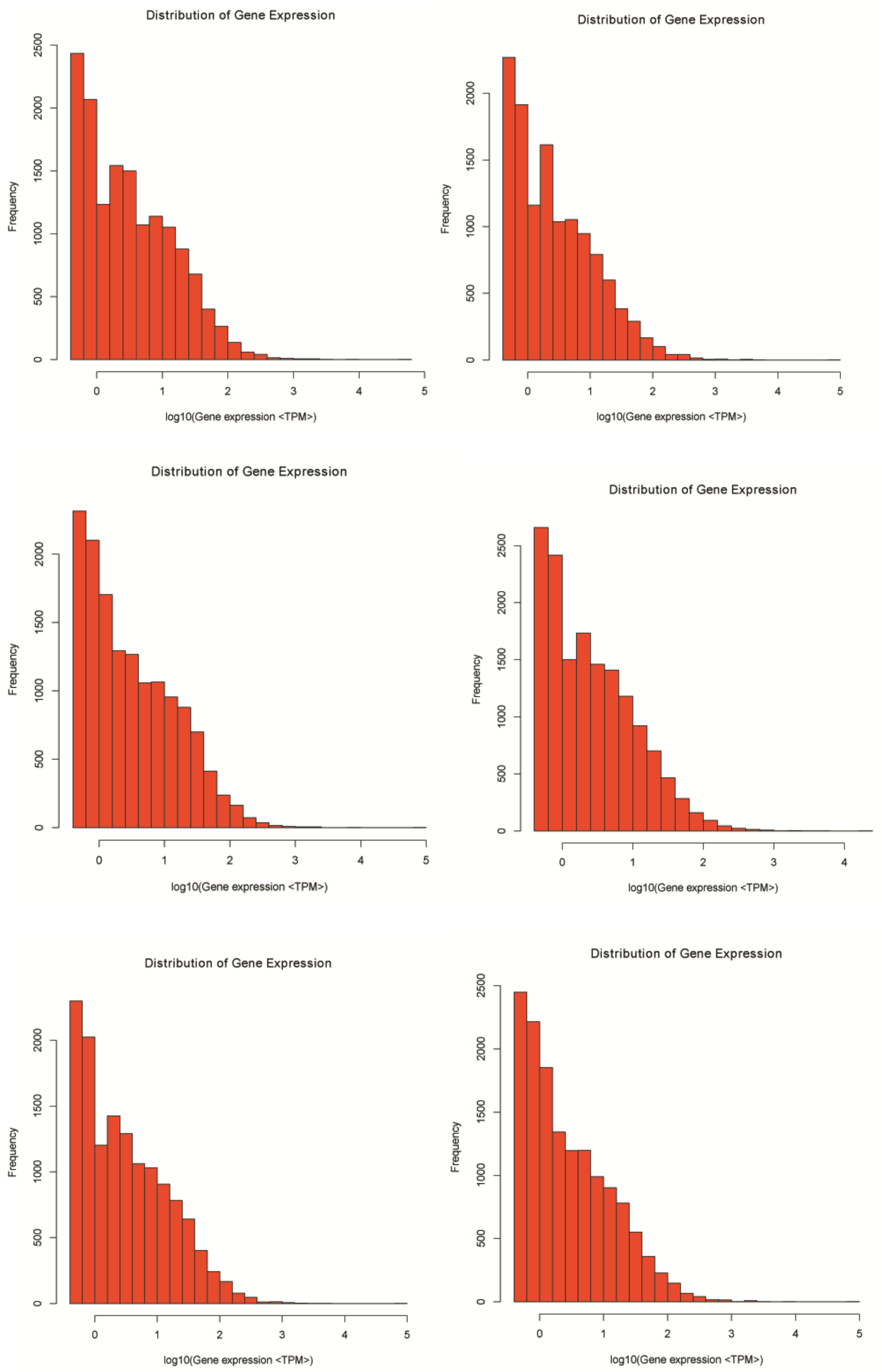


Figure. The gene expression level. TPM (Transcripts Per Million clean tags) is a standardized indicator, pointing out number of transcript copies in every 1 million clean tags. The number of unambiguous clean tags for each gene was calculated and then normalized to TPM.
