# Supplementary material for: De novo characterization of the Anthurium transcriptome and analysis of its digital gene expression under cold stress
Source: BMC Genomics. 2013 Nov 25;14(1):827. doi: 10.1186/1471-2164-14-827 (PMC4046746; doi:10.1186/1471-2164-14-827)
Supplement: Supplementary file 8 — Additional file 8: Significantly enriched pathways by 1-h cold stress in Anthurium. (A) Significantly enriched pathways of differentially expressed genes by 1-h cold stress in Anthurium. (B) Detailed information of photosynthesis pathway in KEGG database. (PDF 248 KB) [file 12864_2013_5516_MOESM8_ESM.pdf]

(A) Significantly enriched pathways of differentially expressed genes by 1-h cold stress in *Anthurium*

Pathway	DEGs with pathway annotation (721)	DEGs with pathway annotation (721)	Pvalue	Qvalue	Pathway ID
Photosynthesis	15 (2.08%)	65 (0.45%)	5.05E-07	5.65E-05	ko00195
Oxidative phosphorylation	22 (3.05%)	214 (1.48%)	0.001032	5.78E-02	ko00190

(B)

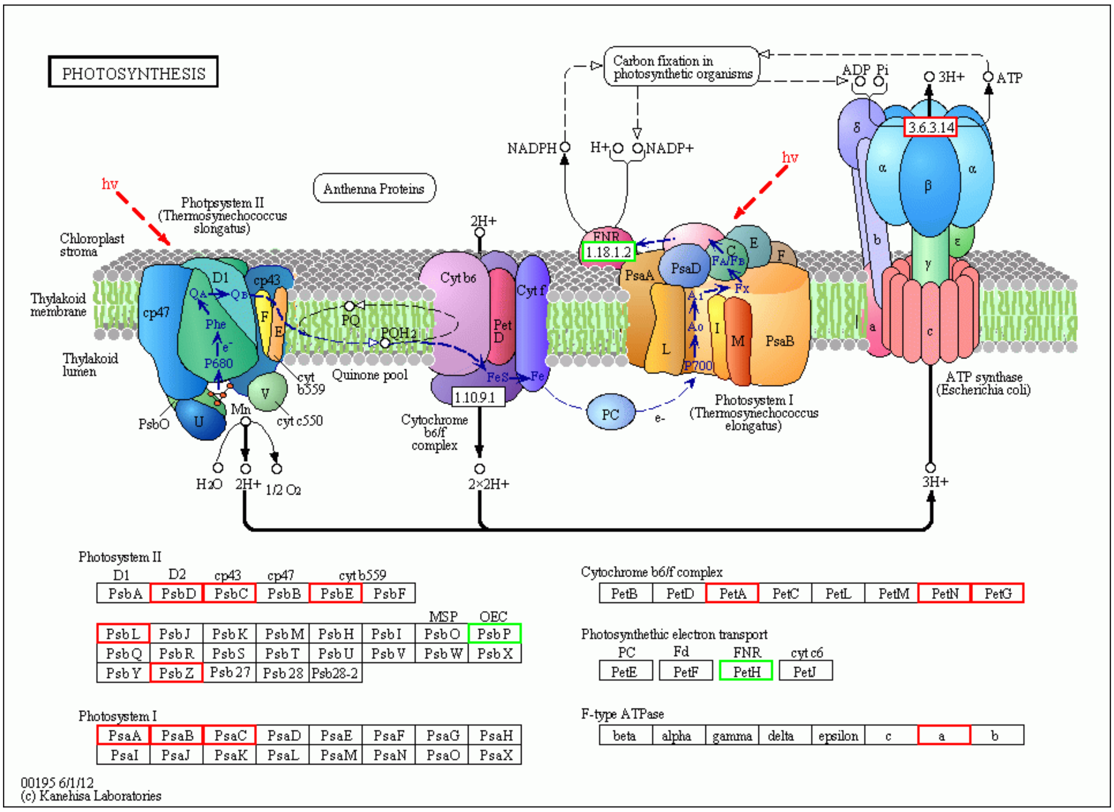

Detailed information of photosynthesis pathway in KEGG database. In the figure, up-regulated genes are marked with red borders while down-regulated genes are marked with green borders. Non-change genes are marked with black borders.
